# The Price of Redo Mitral Valve Replacement: Determinants of Major Adverse Postoperative Events

**DOI:** 10.3390/jcdd13090431

**Published:** 2026-09-02

**Authors:** Barış Timur, Zinar Apaydın

**Affiliations:** Department of Cardiovascular Surgery, Istanbul Mehmet Akif Ersoy Thoracic and Cardiovascular Surgery Training and Research Hospital, İstanbul 34303, Turkey; dr.zinarapaydin@gmail.com

**Keywords:** mitral valve, tricuspid valve, reoperation, postoperative complications

## Abstract

**Background:** Redo mitral valve replacement is a high-risk operation in which early mortality alone may underestimate postoperative burden. We evaluated whether concomitant tricuspid valve repair independently increases major adverse postoperative events after redo mitral valve replacement. **Methods:** Consecutive patients undergoing redo mitral valve replacement between 2019 and 2025 were retrospectively reviewed. Isolated redo mitral valve replacement was compared with redo mitral valve replacement plus concomitant tricuspid valve repair. The primary endpoint was major adverse postoperative events, defined as early mortality, acute kidney injury, cerebrovascular event, prolonged ventilation, reoperation, deep sternal infection, mechanical circulatory support, or permanent pacemaker implantation. **Results:** The final cohort included 249 patients: 134 underwent isolated redo mitral valve replacement and 115 underwent concomitant tricuspid valve repair. Patients receiving tricuspid valve repair were older, had higher pulmonary artery pressure, and had a longer interval from the previous operation. They also had longer ventilation, longer intensive care unit stay and higher rates of atrial fibrillation, atrioventricular block, and permanent pacemaker implantation. Early mortality was similar between groups. Major adverse postoperative events occurred in 122 patients. Concomitant tricuspid valve repair was not associated with major adverse postoperative events in univariable analysis. In multivariable analysis, concomitant tricuspid valve repair was not independently associated with MAPE. Increasing age, smoking, lower left ventricular ejection fraction, and longer cardiopulmonary bypass time remained independently associated with MAPE. **Conclusions:** Early major postoperative morbidity after redo mitral valve replacement was associated primarily with patient-related risk factors and operative complexity. Concomitant tricuspid valve repair was not independently associated with MAPE after adjustment for the available covariates.

## 1. Introduction

Redo mitral valve replacement is one of the most demanding scenarios in contemporary valve surgery. Unlike primary mitral procedures, reoperation is performed in a hostile surgical field shaped by adhesions, distorted atrioventricular anatomy, previous prosthetic material, and progressive cardiopulmonary deterioration. Although experienced centers have reported acceptable early results, redo mitral surgery continues to carry a substantial burden of clinically meaningful complications, including mortality, renal failure, prolonged ventilation, stroke, reoperation, and prolonged intensive care dependency [1,2]. Therefore, the key question is not only whether redo mitral valve replacement can be performed safely, but which factors determine major postoperative morbidity after this high-risk operation.

Concomitant tricuspid valve repair adds further complexity to this problem. Current guidelines support tricuspid intervention during left-sided valve surgery in patients with severe secondary tricuspid regurgitation and in selected patients with annular dilatation or right heart failure [3]. This approach is clinically attractive because untreated tricuspid regurgitation may progress after mitral surgery, and late isolated tricuspid surgery is associated with considerable risk. However, the perioperative cost of adding tricuspid repair remains debated. In the Cardiothoracic Surgical Trials Network randomized trial, concomitant tricuspid annuloplasty reduced tricuspid regurgitation progression after mitral surgery, but did not improve survival or quality of life at 2 years, and increased permanent pacemaker implantation [4]. Recent meta-analytic evidence has similarly suggested a reduction in late tricuspid regurgitation progression without a clear operative mortality penalty, while maintaining concern regarding conduction-related morbidity [5].

This uncertainty is particularly relevant in the redo mitral setting, where concomitant tricuspid repair may reflect a more chronic and advanced disease phenotype rather than a simple additional procedure. Available studies frequently combine repair and replacement, include additional cardiac procedures, or focus primarily on mortality. Consequently, the predictors of major early morbidity after isolated redo mitral valve replacement with or without concomitant tricuspid valve repair remain insufficiently defined. In this study, we evaluated a homogeneous cohort of patients undergoing redo mitral valve replacement alone or with concomitant tricuspid valve repair. We aimed to identify the preoperative and operative predictors of major adverse postoperative events and to determine whether concomitant tricuspid repair independently contributes to early postoperative morbidity.

## 2. Materials and Methods

This retrospective observational study was conducted at Istanbul Mehmet Akif Ersoy Thoracic and Cardiovascular Surgery Training and Research Hospital. Consecutive patients who underwent redo mitral valve replacement between 2019 and 2025 were reviewed from the institutional surgical database. The study population was restricted to patients who underwent redo mitral valve replacement either as an isolated procedure or in combination with concomitant tricuspid valve repair. Patients who underwent additional concomitant cardiac procedures, including aortic valve surgery, mitral valve repair, tricuspid valve replacement, coronary artery bypass grafting, aortic surgery, Bentall procedure, pulmonary valve surgery, atrial septal defect closure, intracardiac mass excision, or other major concomitant procedures, were excluded in order to obtain a homogeneous redo mitral valve replacement cohort.

All procedures were performed through median resternotomy. Mitral valve replacement was performed under aortic cross clamping and cardioplegic arrest. In patients undergoing concomitant tricuspid valve repair, the tricuspid repair was performed during the same period of aortic cross clamping and cardioplegic arrest rather than after removal of the cross clamp or on the beating heart. Tricuspid valve repair was performed using a rigid, incomplete, three-dimensional tricuspid annuloplasty ring. All procedures were performed at the same tertiary cardiovascular surgery center by, or under the direct supervision of, consultant cardiac surgeons experienced in redo valve surgery. All procedures included in the study were performed electively. Patients underwent preoperative cardiological evaluation and were referred for surgery after optimization of medical treatment, volume status, and haemodynamic condition.

All patients were under cardiology follow-up before surgery. Heart failure treatment and diuretic therapy, when clinically indicated, were individualized and managed by the treating cardiology team. Patients underwent surgery after optimization of medical therapy, volume status, and haemodynamic stability.

After application of the inclusion and exclusion criteria, the final analytic cohort consisted of 249 patients. Patients were categorized according to the index surgical procedure. The isolated redo MVR group included patients undergoing redo mitral valve replacement without another valve intervention. The redo MVR+TVP group included patients undergoing redo mitral valve replacement with concomitant tricuspid valve repair and no other major concomitant cardiac procedure.

The indication for concomitant tricuspid valve repair was based on an integrated assessment of preoperative echocardiographic, clinical, and intraoperative findings. Repair was generally considered in patients with at least moderate tricuspid regurgitation, particularly in the presence of tricuspid annular dilatation, right-sided chamber enlargement, or impaired leaflet coaptation. In selected patients with a lower grade of tricuspid regurgitation, repair was considered when substantial annular dilatation or inadequate leaflet coaptation was confirmed intraoperatively. Pulmonary artery pressure was considered an indicator of the overall right-sided haemodynamic burden, but was not used as an isolated indication. No single prospectively defined indexed annular diameter or pulmonary artery pressure threshold was applied throughout the retrospective study period. The final indication was based on the combined preoperative and intraoperative findings.

All patients had provided written general consent at the time of hospitalization and/or surgery for the use of their anonymized clinical data for scientific purposes. Because of the retrospective design of the present study and the use of anonymized data, the requirement for additional study-specific informed consent was waived. The study was conducted in accordance with the principles of the Declaration of Helsinki.

Demographic, clinical, echocardiographic, operative, and postoperative data were obtained from medical records, operative reports, anesthesia charts, intensive care unit records, and the institutional database. Preoperative variables included age, sex, body mass index, hypertension, diabetes mellitus, chronic obstructive pulmonary disease, smoking status, history of myocardial infarction, previous cerebrovascular event, chronic kidney disease, left ventricular ejection fraction, pulmonary artery pressure, heart failure, New York Heart Association functional class, type of previous cardiac surgery, number of redo operations, and the interval between the previous operation and the index redo procedure.

Operative variables included the type of index operation, use of femoral or jugular cannulation, intraoperative injury, operative urgency, infective endocarditis, cardiopulmonary bypass time, and aortic cross clamp time. Postoperative variables included mechanical ventilation duration, intensive care unit stay, total hospital stay, reoperation for bleeding or tamponade, gastrointestinal complications, acute kidney injury, respiratory complications, cerebrovascular events, inotropic support, rhythm disturbances, atrioventricular block, permanent pacemaker implantation, intra-aortic balloon pump use, extracorporeal membrane oxygenation requirement, mediastinitis, wound infection, and early mortality.

The primary endpoint was major adverse postoperative events. This composite endpoint was defined a priori using the Society of Thoracic Surgeons operative mortality and major morbidity domains as a clinical framework, and was modified for the specific context of redo mitral valve surgery [6,7]. Major adverse postoperative events were defined as the occurrence of at least one of the following events: early mortality, acute kidney injury, postoperative cerebrovascular event, prolonged mechanical ventilation exceeding 24 h, reoperation for bleeding or tamponade, mediastinitis or deep sternal wound infection, need for intra-aortic balloon pump or extracorporeal membrane oxygenation, and permanent pacemaker implantation.

Patients who experienced at least one of these complications were classified as MAPE positive. Patients without any of these events were classified as MAPE negative. Postoperative atrial fibrillation, transient conduction disturbances, inotropic support, and intensive care unit length of stay were analyzed as secondary postoperative outcomes and were not included in the composite endpoint.

Early mortality was defined as in-hospital mortality or death within 30 days after surgery. Acute kidney injury was defined according to modified kidney disease: Improving Global Outcomes serum creatinine criteria as an increase in postoperative serum creatinine to at least 1.5 times the baseline value or the requirement for renal replacement therapy during the postoperative hospitalization. Prolonged mechanical ventilation was defined as invasive mechanical ventilation lasting more than 24 h after surgery. Cerebrovascular event was defined as a new postoperative focal neurological deficit confirmed by neurological evaluation and imaging when available. Respiratory complications included pneumonia, reintubation, tracheostomy, acute respiratory distress syndrome, or clinically significant pulmonary complications requiring treatment. Permanent pacemaker implantation was defined as postoperative implantation of a permanent pacing device for persistent conduction disturbance.

### Statistical Analysis

Continuous variables were expressed as median with interquartile range, and categorical variables were expressed as number and percentage. The distribution of continuous variables was assessed using visual and analytical methods. Continuous variables were compared using the Mann–Whitney U test. Categorical variables were compared using the Chi-squared test or Fisher exact test, as appropriate.

First, preoperative, operative, and postoperative characteristics were compared between patients undergoing redo mitral valve replacement alone and those undergoing redo mitral valve replacement with concomitant tricuspid valve repair. Second, patients were compared according to MAPE status. Univariable logistic regression analysis was performed to identify variables associated with MAPE. Variables with clinical relevance and variables showing an association with MAPE in univariable analysis were considered for the multivariable logistic regression model. Results were reported as odds ratios with 95% confidence intervals. Concomitant tricuspid valve repair was the primary exposure of interest and was therefore included in the multivariable model irrespective of its univariable statistical significance. The final multivariable model included nine predictor variables. A total of 122 patients experienced MAPE, corresponding to approximately 13.6 events per predictor variable. The model was kept parsimonious to reduce the risk of overfitting. MAPE was analyzed as a binary composite outcome. Patients experiencing more than one MAPE component were counted once and classified as MAPE-positive. A two-sided *p* value below 0.05 was considered statistically significant. Statistical analyses were performed using R software, version 4.5.2 (R Foundation for Statistical Computing, Vienna, Austria).

## 3. Results

### 3.1. Preoperative Characteristics

A total of 249 patients were included in the final analysis. Of these, 134 patients underwent isolated redo mitral valve replacement and constituted the isolated redo MVR group, whereas 115 patients underwent redo mitral valve replacement with concomitant tricuspid valve repair and constituted the redo MVR+TVP group. Preoperative characteristics are summarized in Table 1.

Patients in the redo MVR+TVP group were significantly older than those in the isolated redo MVR group, with a median age of 59.0 years (IQR, 48.0 to 66.0) versus 55.0 years (IQR, 40.0 to 64.0) (*p* = 0.031), respectively. Female sex was more frequent in the redo MVR+TVP group (70% vs. 50%, *p* = 0.002). Chronic kidney disease was also more common among patients undergoing concomitant tricuspid valvuloplasty (TVP) (30% vs. 19%, *p* = 0.044). Pulmonary artery pressure was significantly higher in redo MVR+TVP group, with a median value of 43.0 mmHg (IQR, 35.0 to 55.0) compared with 40.0 mmHg (IQR, 30.0 to 50.0) in isolated redo MVR group (*p* = 0.023). In contrast, left ventricular ejection fraction was similar between groups, with a median value of 55.0% in both groups (*p* = 0.4).

Smoking was more frequent in isolated redo MVR group (42% vs. 29%, *p* = 0.032), and previous cerebrovascular event was also more common in this group (16% vs. 3.5%, *p* < 0.001). The prevalence of hypertension, diabetes mellitus, chronic obstructive pulmonary disease, previous myocardial infarction, heart failure, and New York Heart Association functional class did not differ significantly between groups. The interval between the previous cardiac operation and the index redo procedure was markedly longer in redo MVR+TVP group, with a median interval of 138.0 months (IQR, 72.0 to 240.0) compared with 60.0 months (IQR, 13.0 to 132.0) in isolated redo MVR group (*p* < 0.001). The proportion of patients undergoing a second redo operation was numerically higher in redo MVR+TVP group, although this difference did not reach statistical significance (11% vs. 5.2%, *p* = 0.078).

### 3.2. Operative Characteristics

Operative characteristics are presented in Table 2. Femoral cannulation was used in 54% of patients in redo MVR+TVP group and 44% of patients in the isolated mitral valve replacement (MVR) group (*p* = 0.12). Jugular cannulation was also numerically more frequent in redo MVR+TVP group (34% vs. 25%, *p* = 0.14). Intraoperative injury occurred only in redo MVR+TVP group (3.5% vs. 0%, *p* = 0.044).

Infective endocarditis was numerically more frequent in the isolated redo MVR group (21% vs. 12%), but this difference did not reach statistical significance (*p* = 0.067). Cardiopulmonary bypass time was comparable between groups, with a median value of 135.0 min (IQR, 108.0 to 167.0) in the isolated redo MVR group and 129.0 min (IQR, 111.0 to 174.0) in redo MVR+TVP group (*p* > 0.9). Aortic cross clamp time was also similar between groups (79.5 min vs. 80.0 min, *p* = 0.7).

### 3.3. Postoperative Outcomes and Complications

Postoperative outcomes and complications are summarized in Table 3. Patients undergoing concomitant TVP had a significantly longer postoperative recovery course. Median mechanical ventilation time was 1.0 day in both groups, but the distribution was significantly wider in the redo MVR+TVP group (IQR, 1.0 to 5.0) than in isolated redo MVR group (IQR, 1.0 to 2.0) (*p* = 0.003). Median intensive care unit stay was also significantly longer in redo MVR+TVP group (4.0 days; IQR, 2.0 to 9.0) compared with the isolated redo MVR group (2.0 days; IQR, 1.0 to 5.0) (*p* < 0.001). Total hospital stay did not differ significantly between groups (12.0 days vs. 11.0 days, *p* = 0.2).

Several major postoperative complications were numerically more frequent after MVR+TVP, although these differences were not statistically significant. Reoperation for bleeding or tamponade occurred in 22% of redo MVR+TVP group and 14% of isolated redo MVR group (*p* = 0.12). Acute kidney injury occurred in 33% and 25% of patients, respectively (*p* = 0.2). Postoperative respiratory complications were observed in 30% of patients in redo MVR+TVP group and 24% of patients in the isolated redo MVR group (*p* = 0.2). Postoperative cerebrovascular events occurred in 7.0% and 4.5% of patients, respectively (*p* = 0.4).

Concomitant TVP was associated with a significantly higher rhythm and conduction burden. Inotropic support was also required more frequently in redo MVR+TVP group (69% vs. 54%, *p* = 0.016). Postoperative rhythm status differed significantly between groups (*p* = 0.001). Normal sinus rhythm was observed in 37% of redo MVR+TVP group compared with 60% of isolated redo MVR group, whereas atrial fibrillation was observed in 57% and 37% of patients, respectively. Atrioventricular block was more frequent in the MVR+TVP group (15% vs. 6.7%, *p* = 0.038). Permanent pacemaker implantation was also more common after MVR+TVP (11% vs. 5.2%, *p* = 0.027). The use of intra-aortic balloon pump, extracorporeal membrane oxygenation, mediastinitis, and wound infection rates were similar between groups. Early mortality was 17% in the MVR+TVP group and 13% in the isolated redo MVR group, without a statistically significant difference (*p* = 0.4).

### 3.4. Baseline Characteristics According to MAPE Status

Patients were subsequently stratified according to the occurrence of major adverse postoperative events. Baseline characteristics according to MAPE status are presented in Table 4. Overall, 122 patients developed MAPE, whereas 127 patients did not. Patients with MAPE were significantly older than those without MAPE, with a median age of 61.0 years (IQR, 52.0 to 68.0) compared with 51.0 years (IQR, 38.0 to 61.0) (*p* < 0.001). Male sex was more frequent in the MAPE group (49% vs. 33%), whereas female sex was more frequent in the No MAPE group (67% vs. 51%) (*p* = 0.010).

Patients who developed MAPE had a higher burden of comorbidity. Hypertension was more frequent in the MAPE group (48% vs. 35%, *p* = 0.039), as were chronic obstructive pulmonary disease (22% vs. 8.7%, *p* = 0.003), smoking history (44% vs. 28%, *p* = 0.006), previous myocardial infarction (8.2% vs. 0.8%, *p* = 0.004), chronic kidney disease (37% vs. 11%, *p* < 0.001), and heart failure (25% vs. 15%, *p* = 0.040). Diabetes mellitus was numerically more frequent in patients with MAPE (31% vs. 21%), but this did not reach statistical significance (*p* = 0.076). Previous cerebrovascular event was similar between groups (9.8% vs. 11%, *p* = 0.8).

Patients with MAPE had significantly lower left ventricular ejection fraction and higher pulmonary artery pressure. Median ejection fraction was 50.0% (IQR, 45.0 to 60.0) in the MAPE group and 57.0% (IQR, 50.0 to 60.0) in the No MAPE group (*p* < 0.001). Median pulmonary artery pressure was 45.0 mmHg (IQR, 35.0 to 56.0) in patients with MAPE and 37.0 mmHg (IQR, 30.0 to 45.0) in patients without MAPE (*p* < 0.001). The frequency of concomitant TVP was not significantly different between patients with and without MAPE (51% vs. 42%, *p* = 0.2). The interval between the previous cardiac operation and the index redo procedure was also comparable between groups (96.0 months vs. 108.0 months, *p* = 0.4), as was the number of redo operations (*p* = 0.14).

### 3.5. Univariable Predictors of MAPE

Univariable logistic regression analysis for MAPE is shown in Table 5. Increasing age was associated with a higher risk of MAPE (OR 1.05 per year, 95% CI 1.03 to 1.07, *p* < 0.001). Chronic obstructive pulmonary disease (OR 3.00, 95% CI 1.45 to 6.60, *p* = 0.004), smoking (OR 2.09, 95% CI 1.24 to 3.56, *p* = 0.006), hypertension (OR 1.71, 95% CI 1.03 to 2.86, *p* = 0.039), and heart failure (OR 1.94, 95% CI 1.03 to 3.71, *p* = 0.042) were also associated with MAPE in univariable analysis. Diabetes mellitus showed a borderline association with MAPE (OR 1.68, 95% CI 0.95 to 2.99, *p* = 0.077).

Lower ejection fraction was associated with a higher risk of MAPE, with an OR of 0.96 per 1% increase in ejection fraction (95% CI 0.93 to 0.99, *p* = 0.003). Higher pulmonary artery pressure was also associated with increased risk (OR 1.03, 95% CI 1.01 to 1.05, *p* < 0.001). Longer cardiopulmonary bypass time was associated with MAPE (OR 1.01, 95% CI 1.01 to 1.02, *p* < 0.001), whereas aortic cross clamp time was not significantly associated with MAPE (OR 1.01, 95% CI 1.00 to 1.01, *p* = 0.2). Concomitant TVP was not significantly associated with MAPE in univariable analysis (OR 1.44, 95% CI 0.88 to 2.39, *p* = 0.2).

### 3.6. Multivariable Predictors of MAPE

The multivariable logistic regression model is presented in Table 6. Concomitant tricuspid valve repair was not independently associated with MAPE after adjustment for the available clinical and operative covariates (OR 1.41, 95% CI 0.80–2.53; *p* = 0.20).

Increasing age remained independently associated with MAPE (OR 1.04 per year, 95% CI 1.01–1.06; *p* = 0.004). Smoking was also independently associated with an increased risk of MAPE (OR 1.91, 95% CI 1.04–3.55; *p* = 0.038). Higher left ventricular ejection fraction was independently associated with lower odds of MAPE (OR 0.97 per 1% increase, 95% CI 0.94–0.99; *p* = 0.021), whereas longer cardiopulmonary bypass time was independently associated with increased odds of MAPE (OR 1.01 per minute, 95% CI 1.00–1.02; *p* = 0.002).

Pulmonary artery pressure showed a borderline association but did not reach statistical significance in the adjusted model (OR 1.02 per mmHg, 95% CI 1.00–1.04; *p* = 0.080). Hypertension, diabetes mellitus, and chronic obstructive pulmonary disease were not independently associated with MAPE.

## 4. Discussion

The present study demonstrates that patients undergoing redo mitral valve replacement with concomitant tricuspid valve repair represented a clinically distinct phenotype compared with those undergoing isolated redo mitral valve replacement. Patients in the redo MVR+TVP group were older and had higher pulmonary artery pressure, a greater prevalence of chronic kidney disease, and a markedly longer interval from the previous cardiac operation. They also experienced a more complicated postoperative course, characterized by longer mechanical ventilation and intensive care unit stay, greater need for inotropic support, and more frequent rhythm and conduction disturbances, including permanent pacemaker implantation. However, early mortality was similar between the groups. After adjustment for the available clinical and operative covariates, concomitant TVP was not independently associated with MAPE. Increasing age, smoking, lower left ventricular ejection fraction, and longer cardiopulmonary bypass time remained independently associated with the composite endpoint. These findings suggest that the observed crude postoperative differences between the treatment groups cannot be attributed solely to the addition of tricuspid valve repair. Nevertheless, because detailed quantitative measurements of tricuspid valve morphology and right ventricular function were not uniformly available, residual confounding related to treatment selection and unmeasured right-sided disease severity cannot be excluded.

Redo mitral surgery has consistently been described as one of the most challenging domains of contemporary valve surgery. Large contemporary series have demonstrated that outcomes have improved over time, but redo patients still carry higher comorbidity, higher replacement rates, and greater postoperative morbidity than primary mitral surgery patients [8]. Recent redo mitral and redo valve series continue to report meaningful early mortality and morbidity despite modern perioperative care, supporting the idea that survival alone underestimates the clinical burden of these procedures [1,2,9,10]. This is the reason we used a composite endpoint based on operative mortality and major morbidity domains rather than early mortality alone. The STS framework identifies major complications such as stroke, renal failure, prolonged ventilation, deep sternal wound infection, and reoperation as central quality outcomes after cardiac surgery [6,7]. In the redo mitral setting, this approach is particularly relevant because a patient may survive the operation, but still experience a clinically costly recovery dominated by renal injury, prolonged ventilation, neurological events, mechanical circulatory support, or permanent pacing.

The role of concomitant TVP during mitral surgery remains one of the most debated issues in valve surgery. Both ACC/AHA and ESC/EACTS guidelines support tricuspid intervention during left-sided valve surgery in patients with severe secondary TR and in selected patients with annular dilatation or right heart failure features [3,11]. The rationale is strong because untreated functional TR may progress after mitral surgery, and delayed isolated tricuspid surgery has historically been associated with substantial risk. However, the perioperative trade off is not negligible. In the CTSN randomized trial, concomitant tricuspid annuloplasty reduced TR progression after surgery for degenerative mitral regurgitation, but did not improve survival or quality of life at 2 years and increased permanent pacemaker implantation [4]. Large observational data from Badhwar et al. suggested that concomitant TV repair at the time of mitral operations was not associated with increased risk adjusted operative mortality [12]. Recent meta-analytic evidence similarly indicates that concomitant tricuspid repair may reduce TR progression without a clear operative mortality penalty, although concerns persist regarding conduction-related morbidity and pacemaker implantation [5,13]. Our findings are broadly aligned with the literature: TVP increased the apparent postoperative burden in unadjusted comparisons, especially rhythm and conduction events, but did not independently predict the composite major morbidity endpoint.

The most plausible explanation is that concomitant TVP in redo MVR is not simply an additional suture line or an added procedural step. It identifies a different disease trajectory. Patients in the TVP group had a much longer interval from the previous operation, which likely reflects the slow evolution of secondary TR through persistent pulmonary hypertension, atrial fibrillation, right atrial enlargement, tricuspid annular dilatation, and progressive right-sided remodeling after the initial left-sided valve procedure. This chronicity explains why TVP patients may arrive at redo surgery with a more advanced cardiopulmonary substrate even when operative times are not longer. Contemporary studies on surgical decision-making for concomitant TV repair emphasize that TR grade, annular dimensions, right heart status, comorbidity, and surgeon preference all shape whether TVP is performed [14]. Therefore, the higher postoperative burden in our TVP group should not be interpreted as evidence that tricuspid repair is harmful per se. Rather, it likely reflects a selection process in which TVP is performed in patients who have already declared more advanced right-sided disease.

Our rhythm and conduction findings deserve specific attention. In the present cohort, postoperative AF, AV block, and permanent pacemaker implantation were more frequent after MVR+TVP. This pattern is biologically plausible because tricuspid annuloplasty is performed close to the atrioventricular node and conduction tissue, and redo surgery adds scarring, adhesions, and altered anatomy to the conduction risk. The CTSN trial reported a higher pacemaker rate in patients receiving concomitant tricuspid annuloplasty [4]. More recent studies focusing on pacemaker implantation after combined mitral and tricuspid surgery and after tricuspid valve operations confirm that tricuspid procedures are associated with a meaningful risk of permanent pacing, particularly when combined valve surgery, longer bypass time, or more extensive tricuspid intervention is present [15,16]. Our findings fit this signal well. Importantly, transient block and AF should be interpreted differently from permanent pacemaker implantation. AF and transient conduction disturbances reflect early postoperative electrophysiological instability, whereas permanent pacemaker implantation represents a durable complication with long-term implications. This supports analyzing rhythm outcomes separately while including permanent pacing within a clinically meaningful modified MAPE definition.

The independent predictors of MAPE in our study also support the concept that patient reserve is more important than TVP itself. Older age likely integrates frailty, lower physiological reserve, vascular stiffness, pulmonary vulnerability, and reduced tolerance to prolonged perfusion. Recent valve surgery data confirm that AKI after heart valve surgery is associated with adverse outcomes, and prolonged CPB time has been repeatedly identified as a predictor of severe postoperative renal injury [17]. Studies from Journal of Clinical Medicine have similarly shown that perioperative renal dynamics after cardiac surgery with CPB can predict AKI, and that longer CPB duration is linked to worse renal and intensive care outcomes [18,19]. In this context, CKD should not be viewed as a single comorbidity, but as a marker of limited systemic reserve. A redo MVR patient with CKD, lower EF, and pulmonary hypertension is already standing close to the edge before the sternum is opened.

Lower ejection fraction and longer CPB time further clarify the mechanism of morbidity. Reduced EF reflects impaired left ventricular systolic function and may indicate a limited ability to tolerate ischemia-reperfusion injury, volume shifts, pulmonary hypertension, and postoperative low-output physiology. CPB time, on the other hand, is a surrogate for operative complexity rather than merely a clock variable. In redo MVR, longer CPB may reflect adhesiolysis, difficult exposure, prosthesis explantation, annular reconstruction, bleeding control, myocardial protection challenges, or intraoperative instability. This is consistent with the contemporary redo mitral literature emphasizing that improved outcomes depend not only on the indication for surgery, but also on operative strategy, myocardial protection, exposure, cannulation planning, and perioperative management [1,2,8,9,10]. The fact that TVP was not an independent predictor while CPB time remained significant suggests that the real operative hazard is not the tricuspid repair label itself, but the accumulated complexity of the procedure and the limited reserve of the patient undergoing it.

The clinical implication is not to avoid concomitant TVP in redo MVR. Such an interpretation would be too simplistic and potentially harmful. If significant TR or annular dilatation is left untreated, the patient may later face progressive right heart failure or a high-risk isolated tricuspid reoperation. Conversely, the presence of TR should not automatically trigger TVP without considering frailty, renal function, left ventricular systolic function, pulmonary pressure, urgency, and expected operative complexity. The surgical decision should therefore be individualized. Our findings suggest that surgeons should not interpret the higher crude morbidity of MVR+TVP as proof that TVP independently worsens early outcomes. Instead, TVP should be understood as a warning sign that the patient may carry a more chronic right-sided disease phenotype. Preoperative optimization, renal protection, careful assessment of right ventricular function, annular dimensions and TR severity, deliberate cannulation planning, avoidance of unnecessary CPB prolongation, and realistic counseling regarding pacemaker risk should be central parts of decision making.

This study has several limitations. It is retrospective and single-center in design, and the decision to perform TVP was not randomized but based on surgeon judgment, echocardiographic findings, and intraoperative assessment. Therefore, selection bias and confounding by indication cannot be excluded. Detailed quantitative right-heart echocardiographic parameters, including TAPSE, right ventricular fractional area change, right atrial and right ventricular dimensions, tricuspid annular diameter, and standardized classification of the mechanism of tricuspid regurgitation, including atrial functional TR, were not uniformly available throughout the study period. Consequently, these variables could not be incorporated into the adjusted analysis. Although all patients underwent preoperative cardiological evaluation and medical optimization, detailed data regarding individual heart failure medications, drug doses, treatment duration, and perioperative medication changes were not uniformly available. The MAPE endpoint was modified from established operative mortality and major morbidity domains and adapted to redo mitral surgery, which improves clinical relevance but may limit direct comparison with studies using strict STS definitions. Long term follow-up was not available for all patients, preventing robust assessment of late survival, recurrent TR, or late heart failure hospitalization. Because the indication for concomitant tricuspid valve repair was not based on a prospectively standardized quantitative protocol, treatment selection included an element of clinical and intraoperative judgment. Although the decision followed an integrated clinical, echocardiographic, and operative assessment, the absence of uniform numerical thresholds may limit the reproducibility and external generalizability of the treatment-selection process. Nevertheless, the strength of this study lies in its homogeneous redo MVR cohort without major concomitant cardiac procedures, and in the separation of crude postoperative burden from independent predictors of major adverse postoperative events. Overall, our findings support a risk model in which redo MVR morbidity is driven primarily by age, renal vulnerability, ventricular reserve, and operative complexity, while concomitant TVP reflects disease chronicity and right-sided burden rather than acting as an independent determinant of early major morbidity.

## 5. Conclusions

In patients undergoing redo mitral valve replacement, concomitant tricuspid valve repair was associated with a more advanced clinical profile, higher pulmonary artery pressure, longer interval from the previous operation, and greater postoperative rhythm and conduction burden, but it was not an independent determinant of major adverse postoperative events. Early major morbidity was driven primarily by age, chronic kidney disease, reduced left ventricular systolic function, and prolonged cardiopulmonary bypass time. These findings suggest that the true postoperative price of redo mitral valve replacement is paid not simply for adding tricuspid repair, but for operating on patients with reduced biological reserve and greater procedural complexity. Surgical decision-making in this setting should therefore move beyond the binary question of whether to repair the tricuspid valve, and should incorporate renal vulnerability, ventricular function, pulmonary vascular burden, and expected operative complexity into preoperative risk assessment.

## Figures and Tables

**Table 1:** Preoperative characteristics.

Characteristic	Isolated MVR Group N = 134 ^1^	Redo MVR+TVP Group N = 115 ^1^	*p*-Value ^2^
Age	55.0 (40.0–64.0)	59.0 (48.0–66.0)	0.031
Gender			0.002
Female	67 (50%)	80 (70%)	
Male	67 (50%)	35 (30%)	
BMI	26.4 (23.8–29.4)	27.3 (23.9–31.2)	0.13
Htn	54 (40%)	48 (42%)	0.8
DM	34 (25%)	31 (27%)	0.8
COPD	24 (18%)	14 (12%)	0.2
Smoking	56 (42%)	33 (29%)	0.032
CVA	22 (16%)	4 (3.5%)	<0.001
CKD	25 (19%)	34 (30%)	0.044
EF	55.0 (50.0–60.0)	55.0 (45.0–60.0)	0.4
PAP	40.0 (30.0–50.0)	43.0 (35.0–55.0)	0.023
Heart failure	24 (18%)	26 (23%)	0.4
NYHA class			0.6
1	3 (2.2%)	4 (3.5%)	
2	65 (49%)	48 (42%)	
3	54 (40%)	54 (47%)	
4	12 (9.0%)	9 (7.8%)	
Interval from previous surgery (months)	60.0 (13.0–132.0)	138.0 (72.0–240.0)	<0.001

^1^ Median (Q1-Q3); *n* (%); ^2^ Wilcoxon rank sum test; Pearson’s Chi-squared test; Fisher’s exact test; BMI: body mass index, COPD: chronic obstructive pulmonary disease, CKD: chronic kidney disease, CVA: cerebrovascular accident, DM: diabetes mellitus, EF: ejection fraction, Htn: hypertension, PAP: pulmonary artery pressure, NYHA: New York Heart Association.

**Table 2:** Operative characteristics.

Characteristic	Isolated MVR Group N = 134 ^1^	Redo MVR+TVP Group N = 115 ^1^	*p*-Value ^2^
Femoral cannulation	59 (44%)	62 (54%)	0.12
Jugular cannulation	34 (25%)	39 (34%)	0.14
Operative injury	0 (0%)	4 (3.5%)	0.044
IE	28 (21%)	14 (12%)	0.067
CPB time (min)	135.0 (108.0–167.0)	129.0 (111.0–174.0)	>0.9
Cross clamp time (min)	79.5 (63.0–103.0)	80.0 (66.0–98.0)	0.7

^1^ n (%); Median (Q1-Q3); ^2^ Pearson’s Chi-squared test; Fisher’s exact test; Wilcoxon rank sum test; CPB: cardiopulmonary bypass IE: infective endocarditis.

**Table 3:** Postoperative outcomes and complications.

Characteristic	Isolated Redo MVR Group N = 134 ^1^	Redo MVR+TVP Group N = 115 ^1^	*p*-Value ^2^
Ventilation time (days)	1.0 (1.0–2.0)	1.0 (1.0–5.0)	0.003
ICU stay (days)	2.0 (1.0–5.0)	4.0 (2.0–9.0)	<0.001
Discharge time (days)	11.0 (7.0–15.0)	12.0 (7.0–23.0)	0.2
Surgical revision	19 (14%)	25 (22%)	0.12
GIS	4 (3.0%)	5 (4.3%)	0.7
AKI	34 (25%)	38 (33%)	0.2
Respiratory complications	32 (24%)	35 (30%)	0.2
CVA	6 (4.5%)	8 (7.0%)	0.4
Inotropic support	72 (54%)	79 (69%)	0.016
Rhythm			0.001
NSR	81 (60%)	43 (37%)	
AF	50 (37%)	65 (57%)	
2 d block	2 (1.5%)	4 (3.5%)	
3 d block	1 (0.7%)	3 (2.6%)	
AV block	9 (6.7%)	17 (15%)	0.038
Permanent pacemaker			0.027
0	128 (94.8%)	102 (89%)	
1	7 (5.2%)	13 (11%)	
IABP	7 (5.2%)	5 (4.3%)	0.7
ECMO	5 (3.7%)	2 (1.7%)	0.5
Mediastinitis	0 (0%)	1 (0.9%)	0.5
SSI	8 (6.0%)	7 (6.1%)	>0.9
Early mortality	17 (13%)	19 (17%)	0.4

^1^ Median (Q1-Q3); n (%); ^2^ Wilcoxon rank sum test; Pearson’s Chi-squared test; Fisher’s exact test; AF: atrial fibrillation AKI: acute kidney injury, AV: atrioventricular, CVA: cerebrovascular accident, ECMO: extracorporeal membrane oxygenation, IABP: intra-aortic balloon pump, ICU: intensive care unit, GIS: gastrointestinal system, NSR: normal sinus rhythm, SSI: surgical site infection.

**Table 4:** Baseline characteristics according to MAPE.

Characteristic	No MAPE N = 127 ^1^	MAPE N = 122 ^1^	*p*-Value ^2^
Age	51.0 (38.0–61.0)	61.0 (52.0–68.0)	<0.001
Gender			0.010
Female	85 (67%)	62 (51%)	
Male	42 (33%)	60 (49%)	
BMI	26.6 (23.9–29.8)	27.2 (24.0–31.2)	0.2
TVP	53 (42%)	62 (51%)	0.2
Htn	44 (35%)	58 (48%)	0.039
DM	27 (21%)	38 (31%)	0.076
COPD	11 (8.7%)	27 (22%)	0.003
Smoking	35 (28%)	54 (44%)	0.006
CVA	14 (11%)	12 (9.8%)	0.8
EF	57.0 (50.0–60.0)	50.0 (45.0–60.0)	<0.001
PAP	37.0 (30.0–45.0)	45.0 (35.0–56.0)	<0.001
Heart Failure	19 (15%)	31 (25%)	0.040
NYHA class			0.070
1	3 (2.4%)	4 (3.3%)	
2	68 (54%)	45 (37%)	
3	47 (37%)	61 (50%)	
4	9 (7.1%)	12 (9.8%)	
Interval from previous surgery (months)	108.0 (24.0–204.0)	96.0 (24.0–180.0)	0.4

^1^ Median (Q1-Q3); n (%); ^2^ Wilcoxon rank sum test; Pearson’s Chi-squared test; Fisher’s exact test; BMI: body mass index, COPD: chronic obstructive pulmonary disease, CVA: cerebrovascular accident, DM: diabetes mellitus, EF: ejection fraction, Htn: hypertension, PAP: pulmonary artery pressure, NYHA: New York Heart Association, TVP: tricuspid valvuloplasty.

**Table 5:** Univariable logistic regression analysis for MAPE.

Characteristic	N	OR	95% CI	*p*-Value
Age	249	1.05	1.03, 1.07	<0.001
BMI	249	1.04	1.00, 1.10	0.057
TVP	249	1.44	0.88, 2.39	0.2
Htn	249	1.71	1.03, 2.86	0.039
DM	249	1.68	0.95, 2.99	0.077
COPD	249	3.00	1.45, 6.60	0.004
Smoking	249	2.09	1.24, 3.56	0.006
EF	249	0.96	0.93, 0.99	0.003
PAP	249	1.03	1.01, 1.05	<0.001
Heart failure	249	1.94	1.03, 3.71	0.042
CPB time	249	1.01	1.01, 1.02	<0.001
Cross clamp time	249	1.01	1.00, 1.01	0.2

BMI: body mass index, COPD: chronic obstructive pulmonary disease, CI: confidence interval, CPB: cardiopulmonary bypass, DM: diabetes mellitus, EF: ejection fraction, Htn: hypertension, OR: odds ratio, PAP: pulmonary artery pressure, TVP: tricuspid valvuloplasty.

**Table 6:** Multivariable logistic regression analysis for MAPE.

Characteristic	OR	95% CI	*p*-Value
Concomitant tricuspid valve repair Age	1.41 1.04	0.80, 2.53 1.01, 1.06	0.2 0.004
Htn	0.92	0.49, 1.73	0.8
DM	1.04	0.53, 2.06	>0.9
COPD	1.70	0.74, 4.11	0.2
smoking	1.91	1.04, 3.55	0.038
EF	0.97	0.94, 0.99	0.021
PAP	1.02	1.00, 1.04	0.080
CPB time (min)	1.01	1.00, 1.02	0.002

CI = confidence interval, COPD: chronic obstructive pulmonary disease, CPB: cardiopulmonary bypass, DM: diabetes mellitus, EF: ejection fraction, OR = odds ratio.

## Data Availability

The data supporting the findings of this study are not publicly available due to privacy and ethical restrictions. De-identified data may be made available from the corresponding author upon reasonable request and subject to institutional approval.

## Abbreviations

The following abbreviations are used in this manuscript:

ACC/AHA	American College of Cardiology/American Heart Association
AF	Atrial fibrillation
AKI	Acute kidney injury
AV	Atrioventricular
BMI	Body mass index
CI	Confidence interval
COPD	Chronic obstructive pulmonary disease
CPB	Cardiopulmonary bypass
CKD	Chronic kidney disease
CTSN	Cardiothoracic Surgical Trials Network
CVA	Cerebrovascular accident
DM	Diabetes mellitus
ECMO	Extracorporeal membrane oxygenation
EF	Ejection fraction
ESC/EACTS	European Society of Cardiology/European Association for Cardio-Thoracic Surgery
GIS	Gastrointestinal system
HTN	Hypertension
IABP	Intra-aortic balloon pump
ICU	Intensive care unit
IE	Infective endocarditis
IQR	Interquartile range
MAPE	Major adverse postoperative events
MVR	Mitral valve replacement
NSR	Normal sinus rhythm
NYHA	New York Heart Association
OR	Odds ratio
PAP	Pulmonary artery pressure
Q1–Q3	First quartile to third quartile
SSI	Surgical site infection
STS	Society of Thoracic Surgeons
TR	Tricuspid regurgitation
TV	Tricuspid valve
TVP	Tricuspid valvuloplasty

## References

[1] Schumacher K., Marin Cuartas M., de la Cuesta M., Noack T., Kiefer P., Leontyev S., A Borger M., Vollroth M., Misfeld M. (2024). Early and long-term outcomes following redo mitral valve surgery in patients with prior minimally invasive mitral valve surgery. Interdiscip. Cardiovasc Thorac. Surg..

[2] Jobeir B.A., De Vol A.E., Alanazi Z.M., Galzerano D., Jobeir A.A., Alsanei A.M., Alamro B., Alamri M., AlHalees Z.Y., Khaliel F.H. (2024). Outcome of patient undergoing redo mitral valve surgery with incidence rate of mitral valve infective endocarditis. J. Cardiothorac. Surg..

[3] Otto C.M., Nishimura R.A., Bonow R.O., Carabello B.A., Erwin J.P., Gentile F., Jneid H., Krieger E.V., Mack M., McLeod C. (2021). 2020 ACC/AHA guideline for the management of patients with valvular heart disease: A report of the American College of Cardiology/American Heart Association Joint Committee on Clinical Practice Guidelines. J. Thorac. Cardiovasc Surg..

[4] Gammie J.S., Chu M.W.A., Falk V., Overbey J.R., Moskowitz A.J., Gillinov M., Mack M.J., Voisine P., Krane M., Yerokun B. (2022). Concomitant Tricuspid Repair in Patients with Degenerative Mitral Regurgitation. N Engl. J. Med..

[5] Poon S.S., Chan J., Ahmed Y., Aslam U., Cianci V., Sharma S., Kumar P. (2024). Concomitant Tricuspid Valve Ring Annuloplasty During Mitral Valve Surgery Versus Mitral Valve Surgery Alone: A Systematic Review and Meta-Analysis. Heart Lung Circ..

[6] Society of Thoracic Surgeons Performance Measure Descriptions. The Society of Thoracic Surgeons: Chicago, IL, USA. https://www.sts.org/quality-safety/performance-measures/descriptions.

[7] Society of Thoracic Surgeons (2020). Definition of Endpoints: Update 6/27/2020.

[8] Mehaffey H.J., Hawkins R.B., Schubert S., Fonner C., Yarboro L.T., Quader M., Speir A., Rich J., Kron I.L., Ailawadi G. (2018). Contemporary outcomes in reoperative mitral valve surgery. Heart.

[9] Salman J., Franz M., Aburahma K., de Manna N.D., Tavil S., Ali-Hasan-Al-Saegh S., Ius F., Boethig D., Zubarevich A., Schmack B. (2024). Hypothermic Ventricular Fibrillation in Redo Minimally Invasive Mitral Valve Surgery: A Promising Solution for a Surgical Challenge. J. Clin. Med..

[10] Sadeghi P., Hosseinsabet A., Mohseni-Badalabadi R., Jalali A., Vakili-Basir A., Pashang M., Omidi N., Bagheri J., Mehrabanian M. (2025). Short- and mid-term outcomes after redo surgical valve replacement. Eur. J. Med. Res..

[11] Vahanian A., Beyersdorf F., Praz F., Milojevic M., Baldus S., Bauersachs J., Capodanno D., Conradi L., De Bonis M., De Paulis R. (2022). 2021 ESC/EACTS Guidelines for the management of valvular heart disease: Developed by the Task Force for the management of valvular heart disease of the European Society of Cardiology (ESC) and the European Association for Cardio-Thoracic Surgery (EACTS). Rev. Esp. Cardiol..

[12] Badhwar V., Rankin J.S., He M., Jacobs J.P., Furnary A.P., Fazzalari F.L., O’bRien S., Gammie J.S., Shahian D.M. (2017). Performing Concomitant Tricuspid Valve Repair at the Time of Mitral Valve Operations Is Not Associated With Increased Operative Mortality. Ann. Thorac. Surg..

[13] Barua A., Wong N., Jain S., Uzzaman M., Nanjaiah P., Jeeji R., Balacumaraswami L. (2025). Concomitant tricuspid surgery for moderate tricuspid regurgitation improves survival in left-sided valve surgery—A meta-analysis. J. Cardiothorac. Surg..

[14] Gollmann-Tepekoylu C., Berretta P., Gerdisch M., Cresce G.D., Kempfert J., Pitsis A., Van Praet F., Rinaldi M., Wilbring M., Yan T. (2025). Surgical decision-making for concomitant tricuspid valve repair in minimally invasive mitral valve surgery. Eur. J. Cardiothorac. Surg..

[15] Ntinopoulos V., Rodriguez Cetina Biefer H., Dzemali O. (2024). Permanent pacemaker implantation after concomitant mitral and tricuspid valve surgery. Eur. J. Cardiothorac. Surg..

[16] Zaheer S., Holmes S.D., Rodriguez E., Winicki N.M., Larson E., Quinn R., Ailawadi G., Gillinov A.M., Gammie J.S. (2025). Factors Associated With Permanent Pacemaker Placement After Tricuspid Valve Operations. Ann. Thorac. Surg..

[17] Kim H.J., Kim J.K., Kim S.O., Han Y., Kang P., Kim J.B. (2025). Acute kidney injury after heart valve surgery: Incidence and impact on mortality based on serum creatinine and urine output criteria. JTCVS Open.

[18] Anzai A., Takaki S., Yokoyama N., Kashiwagi S., Yokose M., Goto T. (2023). Creatinine Reduction Ratio Is a Prognostic Factor for Acute Kidney Injury following Cardiac Surgery with Cardiopulmonary Bypass: A Single-Center Retrospective Cohort Study. J. Clin. Med..

[19] Lee T.H., Lee C.C., Chen J.J., Fan P.C., Tu Y.R., Yen C.L., Kuo G., Chen S.-W., Tsai F.-C., Chang C.-H. (2021). Assessment of Cardiopulmonary Bypass Duration Improves Novel Biomarker Detection for Predicting Postoperative Acute Kidney Injury after Cardiovascular Surgery. J. Clin. Med..

